# Supplementary material for: Pharmacological Comparative Characterization of REL-1017 (Esmethadone-HCl) and Other NMDAR Channel Blockers in Human Heterodimeric N-Methyl-D-Aspartate Receptors
Source: Pharmaceuticals (Basel). 2022 Aug 13;15(8):997. doi: 10.3390/ph15080997 (PMC9414551; doi:10.3390/ph15080997)
Supplement: Supplementary file 1 [file pharmaceuticals-15-00997-s001.zip › pharmaceuticals-1827745-supplementary.pdf]

Pharmacological Comparative Characterization of REL-1017 (Esmethadone-HCl) and Other NMDAR Channel Blockers in Human Heterodimeric N-Methyl-D-Aspartate Receptors

Ezio Bettini[1,*], Stephen M. Stahl[2,3], Sara De Martin[4], Andrea Mattarei[4], Jacopo Sgrignani[5], Corrado Carignani[1], Selena Nola[1], Patrizia Locatelli[5], Marco Pappagallo[6,7], Charles E. Inturrisi[7], Francesco Bifari[8], Andrea Cavalli[5,9], Andrea Alimonti[10-15], Luca Pani[7,16,17], Maurizio Fava[18], Sergio Traversa[7], Franco Folli[19] and Paolo L. Manfredi[7,*]

Supplementary Figures

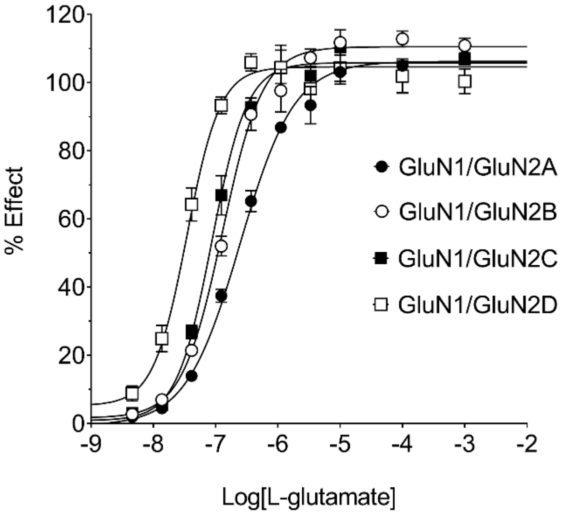

Figure S1. L-glutamate concentration-response curve (CRC) was obtained to characterize the four cell lines expressing different heterodimeric human N-methyl-D-aspartate receptors (NMDARs) by calculating their L-glutamate EC_{50}. L-glutamate CRC was performed with a fluorometric imaging plate reader (FLIPR) assay in the presence of 10 μM glycine and in the absence of Mg^{2+}. L-glutamate CRC included the following 10 final concentrations: 1 mM, 100 μM, 10 μM, 3.3 μM, 1.1 μM, 370 nM, 123 nM, 41 nM, 13.7 nM, and 4.6 nM. L-glutamate EC_{50} was 250 nM, 130 nM, 87 nM, and 34 nM for hGluN1/hGluN2A, hGluN1/hGluN2B, hGluN1/hGluN2C, and hGluN1/hGluN2D receptors, respectively.

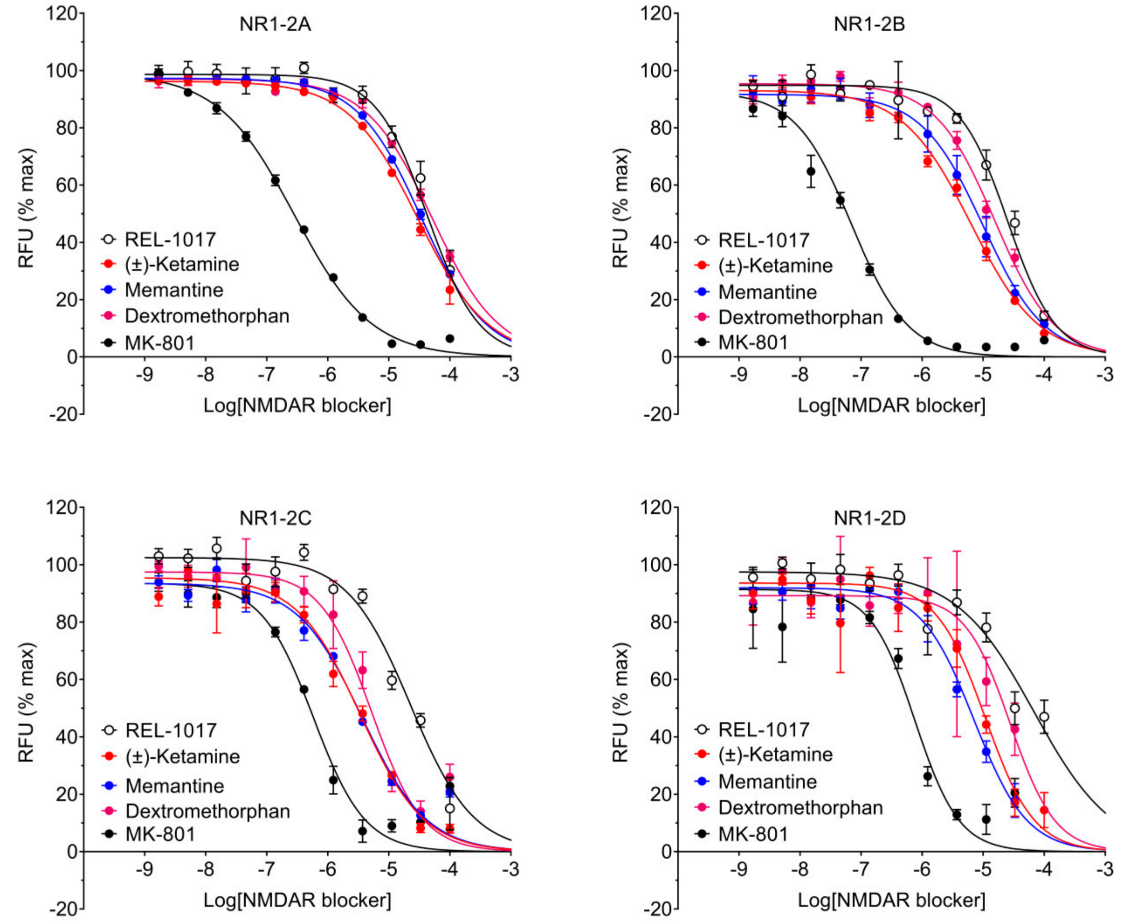

Figure S2. CRCs of REL-1017, (±)-ketamine, memantine, dextromethorphan, and MK-801 were performed in the presence of 10 μM L-glutamate and 10 μM glycine but in the absence of extracellular Mg^{2+} by FLIPR Ca^{2+} assay using Chinese hamster ovary (CHO) cell lines expressing the indicated heterodimeric NMDARs. RFU is relative fluorescence units.

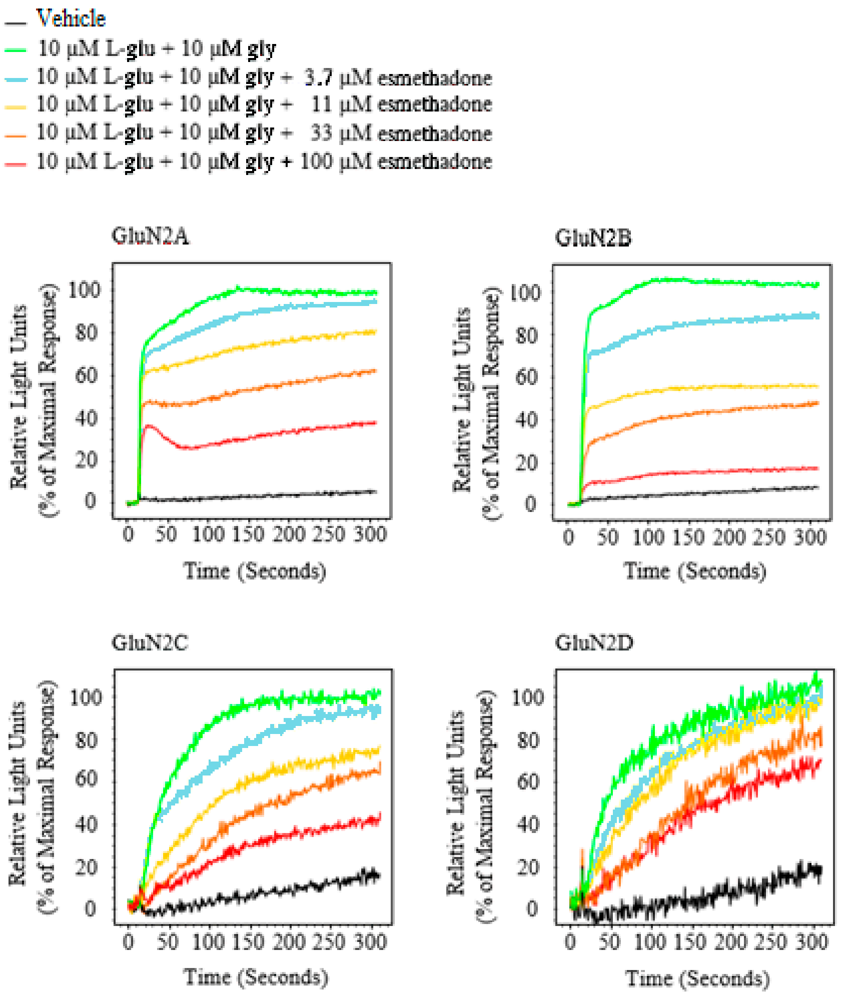

Figure S3. Sample FLIPR traces are shown in presence of vehicle (——), in the presence of 10 µM L-glutamate plus 10 µM glycine alone (——), or in presence of REL-1017 at different concentrations: 3.7 µM (——), 11 µM (——), 33 µM (——), and 100 µM (——) in the four different NMDAR cell lines expressing hGluN1/hGluN2A, hGluN1/hGluN2B, hGluN1/hGluN2C, and hGluN1/hGluN2D receptors. glu is glutamate; gly is glycine.

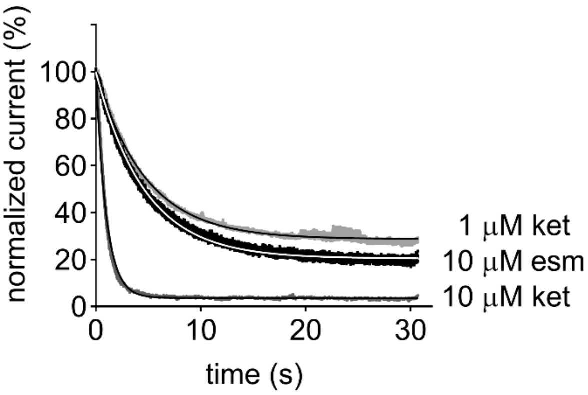

Figure S4. Current values obtained from different electrophysiology experiments during the onset phase were normalized and averaged to derive tau on parameters reported in Table 4. Traces represent % mean ± standard error of current after addition of 10 μM REL-1017 (n = 11), 1 μM (±)-ketamine (n = 10), and 10 μM (±)-ketamine (n = 4), while internal lines are relative fittings. esm is esmethadone; ket is ketamine.

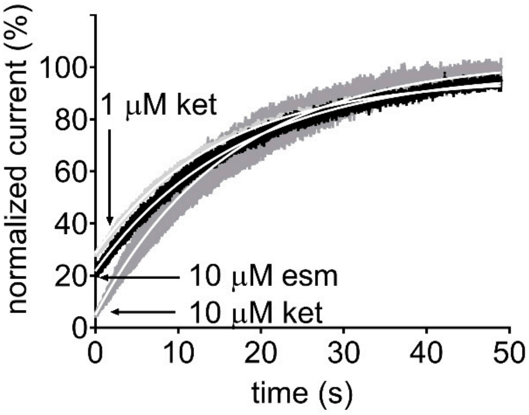

Figure S5. Current values obtained from different experiments during the offset phase were normalized and averaged to derive tau off parameters reported in Table 5. Traces represent % mean ± standard error of current after removal of 10 μM REL-1017 (n = 11), 1 μM (±)-ketamine (n = 10), and 10 μM (±)-ketamine (n = 4), while internal lines are relative fittings. esm is esmethadone; ket is ketamine.

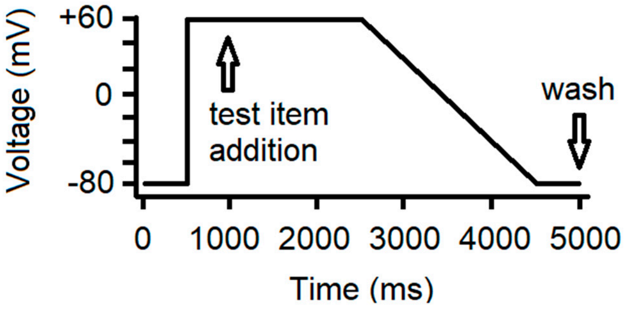

Figure S6. A special protocol was designed to discard cells not perfectly clamped. 1 or 10 µM L-glutamate was added in the presence of 10 µM glycine and 1 mM $MgCl_2$ and in the absence or presence of 10 µM REL-1017 500 milliseconds after a depolarizing step pulse to +60 mV. Cells not perfectly clamped showed a leak current during the first 500 milliseconds of depolarization, even in the absence of L-glutamate, and were not included in analysis. The protocol was repeated three times on each clamped cell. Buffer was added during the first repetition to assess the quality of the seal. L-glutamate was added during the pulse to +60 mV of the second repetition and washed away at the end of the ramp. L-glutamate was added again at the same concentration in the third voltage protocol repetition with (treated group) or without (control group) 10 µM REL-1017. 1 mM $MgCl_2$ was continuously present during L-glutamate addition.

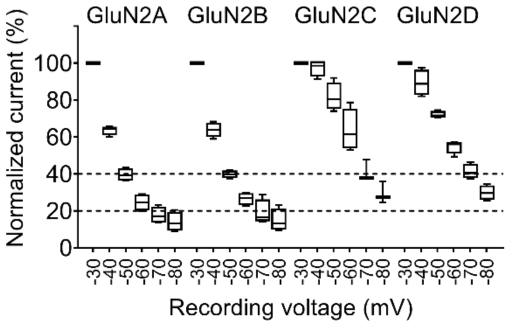

Figure S7. Automated cell clamp was used to verify the magnitude of 1 mM Mg^{2+} blockade at different voltages in cell lines expressing different heterodimeric NMDARs. NMDAR currents decreased at negative voltages in the presence of Mg^{2+} as the ion blocks the NMDAR pore at negative voltages. We verified that Mg^{2+} blockade at negative voltages was less pronounced for hGluN1/hGluN2C and hGluN1/hGluN2D compared to hGluN1/hGluN2A and hGluN1/hGluN2B by normalizing currents recorded at various negative voltages to current recorded at -30 mV. All currents were elicited by 10 μM L-glutamate and 10 μM glycine in the presence of 1 mM $MgCl_2$ (n = 4 for each cell line). Percent current at -60 mV resulted in mean changes of 24 ± 2.4%, 26 ± 1.8%, 64 ± 5.6%, and 55 ± 1.8% (mean ± standard error of the mean [SEM], n = 4) for hGluN1/hGluN2A, hGluN1/hGluN2B, hGluN1/hGluN2C, and hGluN1/hGluN2D, respectively. Data are shown as min to max box plot with median line (n = 4).

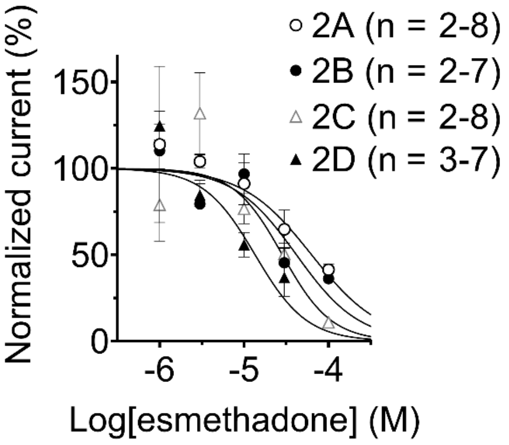

Figure S8. Recordings occurred at -60 mV fixed voltage equal to holding potential. Recordings were obtained in the presence of non-saturating 1 μM L-glutamate, 10 μM glycine, and 1 mM $MgCl_2$ at the end of a 120-second incubation period with L-glutamate and REL-1017. Data are presented as mean ± SEM. IC_{50} and Hill slope values of each fitting are reported in Table 1. The graph represents % current recorded in the presence of 1 μM, 3 μM, 10 μM, 30 μM, or 100 μM REL-1017 and normalized with respect to control and relative fittings in each NMDAR cell line. Fitting values are reported in Table 3.

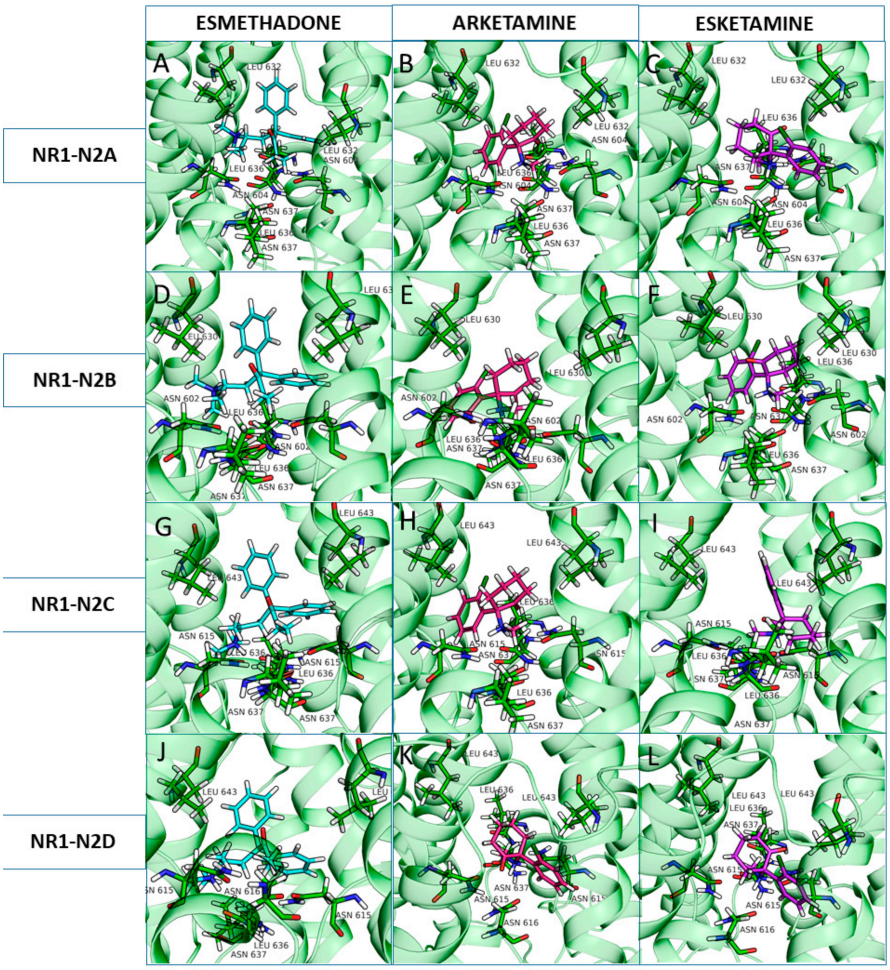

Figure S9

Figure S9. Structures of the complexes between esmethadone (light blue, panels A, D, G, J), arketamine (magenta, panels B, E, H, K), and esketamine (purple, panels C, F, I, L) in NR1-N2C, NR1-N2A, NR1-N2B, and NR1-N2D in the closed conformation models (PDB code 6WHS).

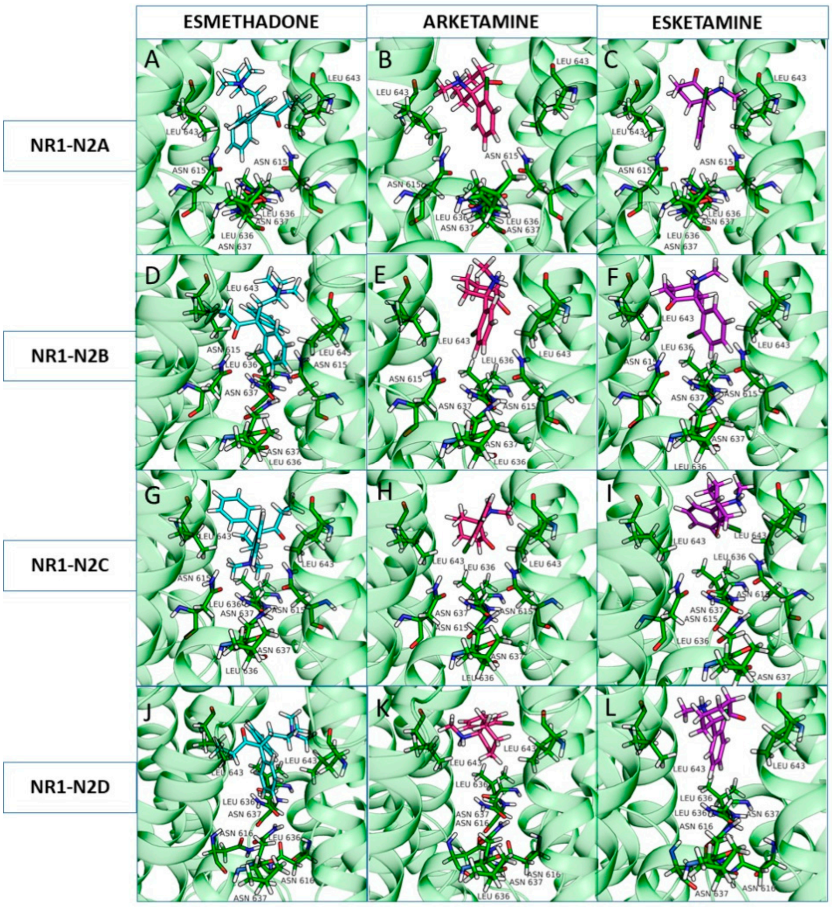

Figure S10

Figure S10. Structures of the complexes between esmethadone (light blue, panels A, D, G, J), arketamine (magenta, panels B, E, H, K), and esketamine (purple, panels C, F, I, L) in the open conformation NR1-N2C model (PDB code 6WHS).

Supplementary Tables

	GluN1/GluN2A			GluN1/GluN2B			GluN1/GluN2C			GluN1/GluN2D		
	K_B (μM)	Affinity ratio (%)	α	K_B (μM)	Affinity ratio (%)	α	K_B (μM)	Affinity ratio (%)	α	K_B (μM)	Affinity ratio (%)	α
REL-1017	8.9	51	0.22	6.1	74	0.26	4.5	100	0.17	7.8	58	0.22
(±)-Ketamine	4.3	11	0.17	1.1	42	0.14	0.46	100	0.13	1.4	33	0.15
Memantine	3.6	8	0.15	0.58	48	0.094	0.28	100	0.10	0.59	47	0.13
Dextrome-thorphan	9.6	13	0.25	1.9	63	0.13	1.2	100	0.24	6.7	18	0.34
(+)-MK-801	0.11	44	0.87	0.048	100	1.0	0.14	34	0.39	0.15	32	0.36

Table S1. Operational equation parameters for REL-1017 and reference NMDAR blockers.

Estimated K_B and α values for five NMDAR channel blockers were obtained via FLIPR by L-glutamate CRCs, alone or in the presence of six different concentrations of the examined test item. Experiments were conducted for the various test items as exemplified in Figure 2 for REL-1017. An operational equation for allosteric modulators was used to estimate K_B and α values for REL-1017 and other test items, using the formula described in the Materials and Methods section.

Tables S2a-S2e. L-glutamate EC$_{50}$ values with or without REL-1017 and reference NMDAR blockers.

L-glutamate EC$_{50}$ and maximal asymptote (% max) values were obtained in the presence of five selected NMDAR channel blockers in a FLIPR assay, as exemplified for REL-1017 in Figure 2. Fitting values were obtained for each heterodimeric NMDAR using a logistic equation in GraphPad Prism v8.0. Tables S2a-S2e are for L-glutamate data in the presence of REL-1017, (±)-ketamine, memantine, dextromethorphan, and (+)-MK-801, respectively.

Table S2a

	GluN1/GluN2A		GluN1/GluN2B		GluN1/GluN2C		GluN1/GluN2D	
REL-1017 concentration (μM)	EC$_{50}$ (μM)	Max. (%)	EC$_{50}$ (μM)	Max. (%)	EC$_{50}$ (μM)	Max. (%)	EC$_{50}$ (μM)	Max. (%)
0	0.25	106	0.13	111	0.087	106	0.034	105
0.049	0.21	103	0.11	96	0.15	99	0.029	101
0.195	0.23	98	0.14	96	0.15	100	0.030	97
0.781	0.26	103	0.14	93	0.14	95	0.034	101
3.1	0.28	98	0.13	89	0.15	94	0.042	97
12.5	0.38	84	0.18	72	0.21	75	0.071	82
50	0.41	35	0.37	35	0.25	30	0.11	41

Table S2b

	GluN1/GluN2A		GluN1/GluN2B		GluN1/GluN2C		GluN1/GluN2D	
(±)-Ketamine concentration (μM)	EC$_{50}$ (μM)	Max. (%)	EC$_{50}$ (μM)	Max. (%)	EC$_{50}$ (μM)	Max. (%)	EC$_{50}$ (μM)	Max. (%)
0	0.25	106	0.13	111	0.087	106	0.034	105
0.049	0.31	100	0.18	98	0.12	97	0.044	98
0.195	0.28	96	0.18	92	0.16	95	0.047	97
0.781	0.42	97	0.20	80	0.23	80	0.063	93
3.1	0.40	87	0.23	70	0.23	65	0.075	81
12.5	0.44	66	0.23	44	0.21	20	0.12	45
50	0.67	38	0.47	24	0.41	6.2	0.19	7.1

Table S2c

	GluN1/GluN2A		GluN1/GluN2B		GluN1/GluN2C		GluN1/GluN2D	
Memantine concentration (μM)	EC$_{50}$ (μM)	Max. (%)	EC$_{50}$ (μM)	Max. (%)	EC$_{50}$ (μM)	Max. (%)	EC$_{50}$ (μM)	Max. (%)
0	0.25	106	0.13	111	0.087	106	0.034	105
0.049	0.26	95	0.16	88	0.15	92	0.048	94
0.195	0.34	92	0.18	84	0.13	85	0.048	89
0.781	0.47	96	0.25	79	0.24	76	0.068	87
3.1	0.40	83	0.23	64	0.30	49	0.080	59
12.5	0.52	68	0.25	43	0.38	20	0.18	26
50	0.80	36	0.43	19	0.55	11	N.A.	19

N.A. is not applicable.

Table S2d

	GluN1/GluN2A		GluN1/GluN2B		GluN1/GluN2C		GluN1/GluN2D	
Dextromethorphan concentration (μM)	EC_{50} (μM)	Max. (%)	EC_{50} (μM)	Max. (%)	EC_{50} (μM)	Max. (%)	EC_{50} (μM)	Max. (%)
0	0.25	106	0.13	111	0.087	106	0.034	105
0.049	0.26	98	0.20	95	0.11	95	0.031	93
0.195	0.24	92	0.21	92	0.15	97	0.031	91
0.781	0.38	99	0.25	88	0.17	86	0.042	99
3.1	0.34	88	0.21	74	0.19	67	0.056	87
12.5	0.38	79	0.20	57	0.21	26	0.086	59
50	0.70	44	0.59	32	0.16	13	0.16	31

Table S2e

	GluN1/GluN2A		GluN1/GluN2B		GluN1/GluN2C		GluN1/GluN2D	
(+)-MK-801 concentration (μM)	EC_{50} (μM)	Max. (%)	EC_{50} (μM)	Max. (%)	EC_{50} (μM)	Max. (%)	EC_{50} (μM)	Max. (%)
0	0.25	106	0.13	111	0.087	106	0.034	105
0.049	0.20	67	0.098	47	0.11	83	0.031	87
0.195	0.36	53	0.10	22	0.12	67	0.053	74
0.781	1.3	35	0.066	9.5	0.13	33	0.034	40
3.1	2.6	6.1	N.A.	< 5	0.10	12	0.018	5.5
12.5	N.A.	< 5	N.A.	< 5	N.A.	< 5	N.A.	< 5
50	N.A.	< 5	N.A.	< 5	N.A.	< 5	N.A.	< 5

N.A. is not applicable.
